# Exploring Preschoolers' Emotions in Pediatric Dentistry

**DOI:** 10.1055/s-0042-1758784

**Published:** 2023-06-09

**Authors:** Dyani R.N. Wadji, Ririn Despriliani, Arlette Suzy Setiawan

**Affiliations:** 1 Department of Dental Studies, Faculty of Dentistry, Universitas Padjadjaran, Bandung, Indonesia; 2 Faculty of Language and Art, Universitas Negeri Jakarta, Jakarta, Indonesia; 3 Department of Pediatric Dentistry, Faculty of Dentistry, Universitas Padjadjaran, Bandung, Indonesia

**Keywords:** children's emotion, dental care, Facial Expression Emotion Scale (FEES)

## Abstract

**Objective** Emotion is a feeling that someone can feel. Emotions are generally shown through behavior or facial expressions. Emotions are essential for dental treatment because there is a relation between the emotions felt by children and the success of the dental treatment that the dentist will give. This study aimed to describe variables of emotions about dental treatment.

**Materials and Methods** Descriptive analysis using a convenience nonrandom sampling technique was conducted on 58 preschool children aged 3 to 6 years old who came for dental treatment at the Bandung Dental Center in Bandung, Indonesia. The instrument used to ask children how they feel about dental care is a 7-item questionnaire derived from the children's fear survey scale-dental subscale. Meanwhile, the media used by children to respond was a card with facial expressions from the Facial Expression Emotion Scale.

**Results** The results showed that only participants aged 4 responded with one type of emotion (happy), while the other age groups gave various emotional responses. Fear emotion began to appear in the age group of 5 and 6 years and only in girls, while emotions of anger also appeared in girls and only at the age of 5.

**Conclusion** In this study, the emotions that children choose about dental care at the Bandung Dental Center clinic are happy emotions. The emotions of fear and sadness were chosen more by girl participants, while none of the boy participants chose the emotion of fear. This sad and fearful response is associated with invasive dental treatment. Anger was chosen as a child's response dominantly because of the parents' invitation to the dentist.

## Introduction

Children's emotions are unique and complicated to understand because one of the characteristics of children's emotions is that the same behavior shows several types of emotions. 1 A child can express his feelings, but sometimes it takes time and help to identify what emotions he is feeling. 2 The role of parents is needed here to help children find appropriate emotions so that children can recognize the emotions they are feeling and understand the four basic emotions, namely, fear, anger, sadness, and joy. 1 3 4 5

The ability to recognize emotions is included in the basis of emotional intelligence. In contrast to intellectual intelligence/intelligence quotient, emotional quotient (EQ) is an ability that can be learned, not something that has been there since birth. This EQ must be taught from an early age to children so that children have an excellent ability to recognize the emotions they are feeling. 3 4 Emotions are essential for dental care because a study by Kurniawati and Amalia in 2019 showed a link between the emotions children feel and the success of the dental treatment that the dentist will give. 6 Therefore, it is crucial for dentists to know what emotions are being felt by a child to achieve optimal dental and oral care. When a child comes to the dentist for treatment, he expresses all his emotions, such as happiness, anger, sadness, and so on. The emotions most often inferred by parents are dental fear (DF) and dental anxiety. 7 DF is one of the normal emotional reactions to one or more certain stimuli while doing the dental treatment because of fear of dentists, dental nurses, noise and vibrations from dental drills, and pressure from hand instruments during dental treatment. 8 Epidemiological studies have shown that the prevalence of DF in the French ranges between 13.1 and 19.8%, although gender difference is not reported. 9 Dental anxiety is the cause of symptoms of psychological disorders, such as fear and feeling uncomfortable about dental treatment, which can be one of the causes of dental treatment failure. 6 This study aimed to describe children's emotions in dental care aside from fear and anxiety.

## Materials and Methods

This quantitative descriptive study with a survey technique was carried out at the Bandung Dental Center (BDC), a private clinic in Bandung City, Indonesia that developed the theme of “growth and development clinic.” The clinic was chosen because it has implemented an appointment system for its patients and a health protocol that follows the World Health Organization directives for coronavirus disease 2019 so that during a pandemic, children's visits to the dentist do not experience a significant reduction or postponement. 10

### Participant Recruitment

The study population was pediatric patients who visited the BDC during the period March to April 2022. The number of patient visits “year-on-year” in March to April 2021 was 50. So based on the Slovin formula with a 95% confidence level and 5% margin of error, 11 the minimum sample size is 45 children. This study used a nonrandom sampling method, a convenience sampling technique with a questionnaire data collection method. Participants were pediatric patients who were between the ages of 3 and 6 years (preschoolers) who come to the BDC for the first time, both boys and girls. Pediatric patients who have shown disruptive behavior during their visit were excluded.

### Instrument Development

**Fig. 1:**
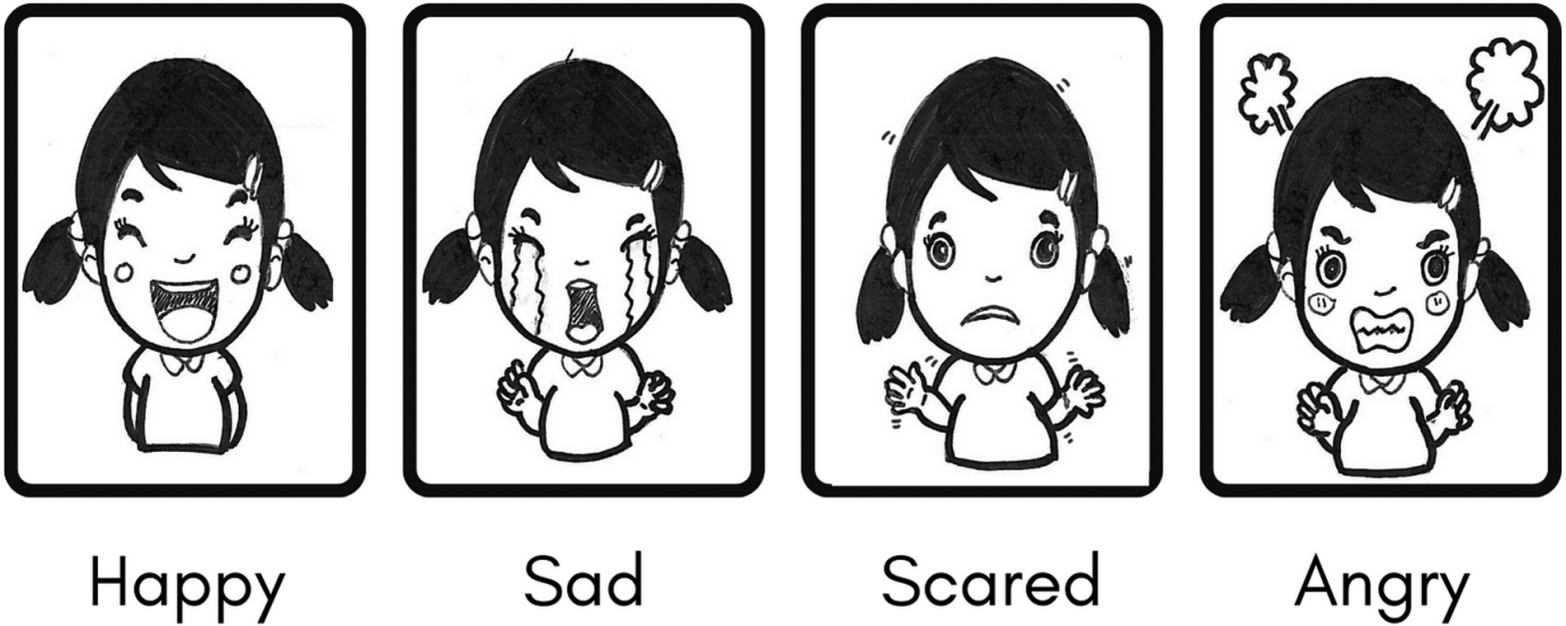
Facial Expression Emotion Scale (FEES). 12

The measuring instrument used is the Facial Expression Emotion Scale (FEES) which consists of four facial images, each of which expresses a primary emotion (happy, sad, afraid, angry) which is printed on a 5R-sized card ( Fig. 1 ). The second and third authors developed this instrument in the previous year. 12

The questions asked to the children included seven questions related to dentists and their treatment. These questions were derived from the questions contained in the Children Fear Survey Scale-Dental Subscale questionnaire. 13 14 The original questionnaire contained 15 questions, but when tested on 30 children for this study, 7 questions turned out to be valid (Cronbach alpha 0.899) with some modification questions, as shown in Table 1 . Each question had the same four answer options; happy, sad, angry, and scared, which the child will answer by showing a card containing an emotional expression.

**Table 1:** Items of questions asked to children

No.	Item	Choice of response			
		**Happy**	**Sad**	**Scared**	**Angry**
1.	How would you feel if your parents took you to the dentist?				
2.	How would you feel if you had to go to the dentist?				
3.	How would you feel if the dentist examined your teeth?				
4.	How would you feel if the dentist cleans your teeth?				
5.	How would you feel if the dentist has to get your tooth filled?				
6.	How would you feel about having your tooth pulled out/extracted?				
7.	How would you feel about having your teeth drilled by the dentist?				

### Research Ethical Aspects

The study has been reviewed and approved by the Health Ethics Commission of the Universitas Padjadjaran (letter number 174/UN6.KEP/EC/2022) and has obtained a research permit from the National Unity Agency and Politics of Bandung (Letter number PP.09.01/168-kesbangpol/III/2022).

### Data Collection

After obtaining informed consent from the parents, the child, still accompanied by one of his/her parents, enters a particular room. The course of the study is explained to the child in a language easily understood by the child—then conditioning the child by showing each facial expression card placed on the table in front of the child. The accompanying parent sitting slightly behind the child, so the parent cannot provide clues for the answer. The investigator sat in front of the child about two meters apart and asked questions according to the assessment sheet. The child is asked to answer by pointing or lifting one of the cards containing the appropriate facial expression. The investigator wrote the children's answers on the questionnaire sheet. Every emotion the child gives through the FEES card is recorded on the participant's answer sheet. The data collection was carried out by two investigators on different children and different periods. Interrater reliability was carried out before the study with a kappa value of 0.9, which means that the reliability of the rater is considered close to perfect. 15

### Analysis

The data from this study are presented in the form of tables and diagrams and are written descriptively.

## Results

The results obtained from the 58 participants follow the established criteria. The distribution of the characteristics of the participants can be seen in Table 2 . Based on Table 2 , the age of most participants is 6 years old, which is 41.3%. There were more girl participants than boy participants, 62.1%. All participants knew what a dentist was and had been to a dentist before (100%).

**Table 2:** Distribution of participant characteristics

Characteristics		*n*	(%)
Age	3-year-old	4	6.9
	4-year-old	12	20.7
	5-year-old	18	31.1
	6-year-old	24	41.3
Gender	Boys	22	37.9
	Girls	36	62.1
Familiar about dentist's work	Yes	58	100
	No	0	0
Dental treatment experience	Yes	58	100
	No	0	0

Overall, the results of this study are shown in Table 3 , which shows that the dominance of emotion is happiness which occupies the highest percentage in each item. At the same time, the emotion with the lowest percentage is anger. When traced by item, it appears that the item “parents took you to the dentist” gives a happy and sad response. Likewise, the item “your teeth being cleaned by the dentist.” The fear response began to appear in items related to invasive procedures such as filling, drilling, and extracting teeth.

**Table 3:** Participants' responses

No.	Item	Choice of response							
		Happy		Sad		Scared		Angry	
		*n*	%	*n*	%	*n*	%	*n*	%
1.	How would you feel if your parents took you to the dentist?	40	69	18	31	0	0	0	0
2.	How would you feel if you had to go to the dentist?	40	69	17	29	0	0	1	2
3.	How would you feel if the dentist examined your teeth?	40	69	16	28	2	3	0	0
4.	How would you feel if the dentist cleans your teeth?	44	78	14	24	0	0	0	0
5.	How would you feel if the dentist has to get your tooth filled?	40	69	12	21	6	10	0	0
6.	How would you feel about having your tooth pulled out/extracted?	40	69	12	21	6	10	0	0
7.	How would you feel about having your teeth drilled by the dentist?	40	69	12	21	5	7	1	2

**Graph 1:**
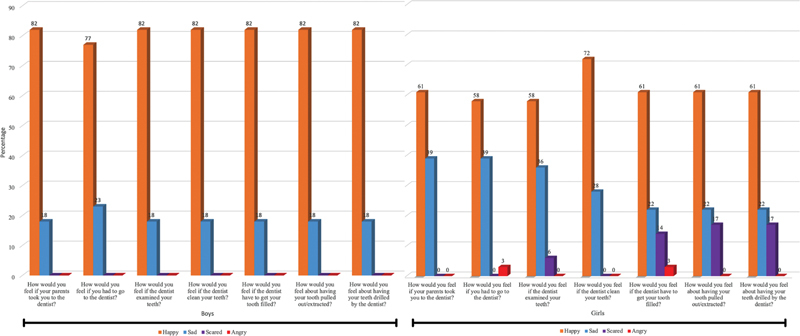
Distribution of emotional responses in grouping by gender.

Responses grouped based on the gender of the participants are presented in Graph 1 . From the graph, it can be seen that the boy participants only gave “happy” and “sad” responses. On the other hand, while girl participants responded to all emotions even though they were the same as boy participants, the dominant responses were “happy” and “sad” emotions.

**Graph 2:**
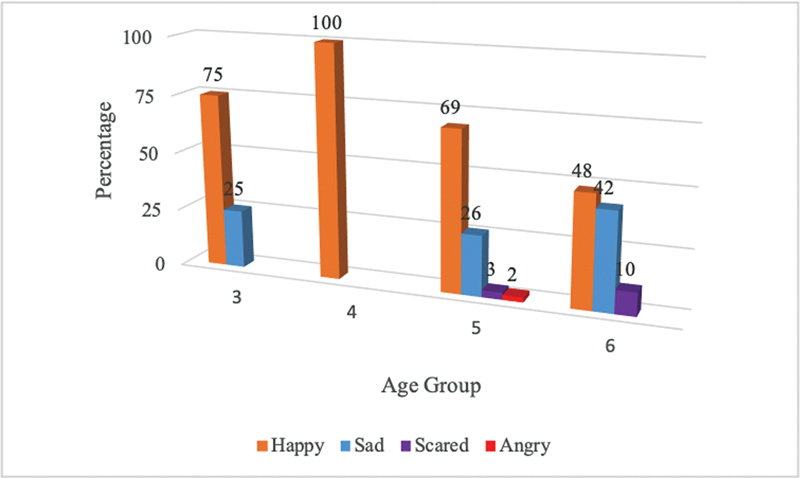
Distribution of emotional responses by age group.

Participants' responses grouped by age are presented in Graph 2 . Here, it can be seen that the “happy” response was given by all age groups, with the highest response in the 4-year age group, where all participants (100%) gave a “happy” response. On the other hand, the “scared” response began to appear in the 5- and 6-year age groups. Meanwhile, the “angry” response only appeared in the 5-year age group.

## Discussion

Most participants who came to the clinic were girls, 62.01%. This is in line with a study conducted by Gigi et al, 16 where the number of dental and oral care patients were more girls than boys; 1,240 girls (63.14%) and 724 boys (36.86%). This can be related to the results of a study that is grouped by gender, where it is seen that girls show more variety of emotions. Gender differences in emotional expression result from biologically based temperament tendencies and socialization of boys and girls who use gender-related rules to display emotional expression. 17

No boy participants chose fear as the response to the question, while girl participants chose the emotion of fear as the response to the emotion they felt while having dental treatment. The study of Rehatta et al supports the results of this report, 18 which showed severe anxiety in 15 girls (27.27%) while only 13 (23.64%) boys felt severe anxiety during dental procedures. This follows the statement of Saputro and Fajrin, as stated by Kurniawati 5 that girls have higher negative emotional feelings than boys, such as crying, fear, anxiety, anger, etc. Girls tend to show more assertive expressions of fear than boys. 1 Sad emotions were also chosen more by girls, with as many as 30 responses (25.21%). This result follows the statement that girls are likelier to feel anxious, impatient, and tear easily. In addition, women tend to be more open in showing what they feel than men, who usually prefer to keep what they feel, and men also have more stable emotions. This can be the reason for the emotional difference between the gender. 18 19

The results of this study indicate that most participants who come to the clinic are 6 years old. This follows the study of Sanger et al, 20 who said that the age period of 6 to 8 years is when children are often associated with dental and oral care because, in that age range, there are many eruptions of permanent teeth starting from the first molar. When associated with the emotional response selected by participants based on age group, in the age group of 3 and 4 years, participants only displayed between “happy” and “sad.” This can be related to the characteristics of participants who already know a dentist and have been to a dentist before. This can affect the emotions felt by participants while doing dental treatment. Because children's behavior in dental and oral care can be influenced by several factors, one of which is a history of previous dental treatment. 21 If the child has had dental and oral care before with a friendly and pleasant dentist, it will significantly affect the child's behavior so that the child becomes cooperative during the treatment. A friendly dentist and a comfortable environment make children not afraid and anxious to undergo dental treatment. 22 This follows the study of Kurniawati and Amalia, 6 which shows that pediatric patients who have had previous treatment are patients who do not feel anxious, study on 44 children (67.7%). Children who visit for the second time or more will have a lower level of dental anxiety because the child has been able to get used to the environment and has good coping with that anxiety. This can happen because dentists can control behavior and recognize children's emotions so that children do not feel afraid and anxious.

Fear of dental treatment or DF, which is often said to be dominant, occurs in children 23 24 contradicts the results of this study. However, this does not necessarily indicate a change in children's emotions due to changing generations or the development of technology and pediatric dentistry. The limitations of this study include the number of participants for each age is not the same because the population only comes from one place. In addition, the clinic used as the study location has been designed to be “child friendly.” Dentists use personal protective equipment with cartoon characters so that it is far from the “white coat effect” that children fear. Maybe further study needs to be done to compare children's emotional descriptions based on a comparable number of samples with more diverse age variations. A similar study needs to be carried out in other places to see if there are other variations in answers. Dentists should understand children's emotions while doing dental treatment so that children can feel comfortable and provide optimal care. Cooperation from parents is also needed so that children can control their emotions while receiving treatment from the dentist. 14

The future benefit of the results of this study is to get an overview of children's emotions in dental care which can be used as a reference for further study. In addition, the data obtained can help clinicians predict the emotions of children who will undergo dental treatment so that they can be of suggestion for handling the emotions for doctors to obtain optimal treatment results.

## Conclusion

In this study, the emotions that children choose about dental care at the BDC clinic are happy emotions. The emotions of fear and sadness were chosen more by girl participants, while none of the boy participants chose the emotion of fear. This sad and fearful response is associated with invasive dental treatment. Anger was chosen as a child's response dominantly because of the parents' invitation to the dentist.
